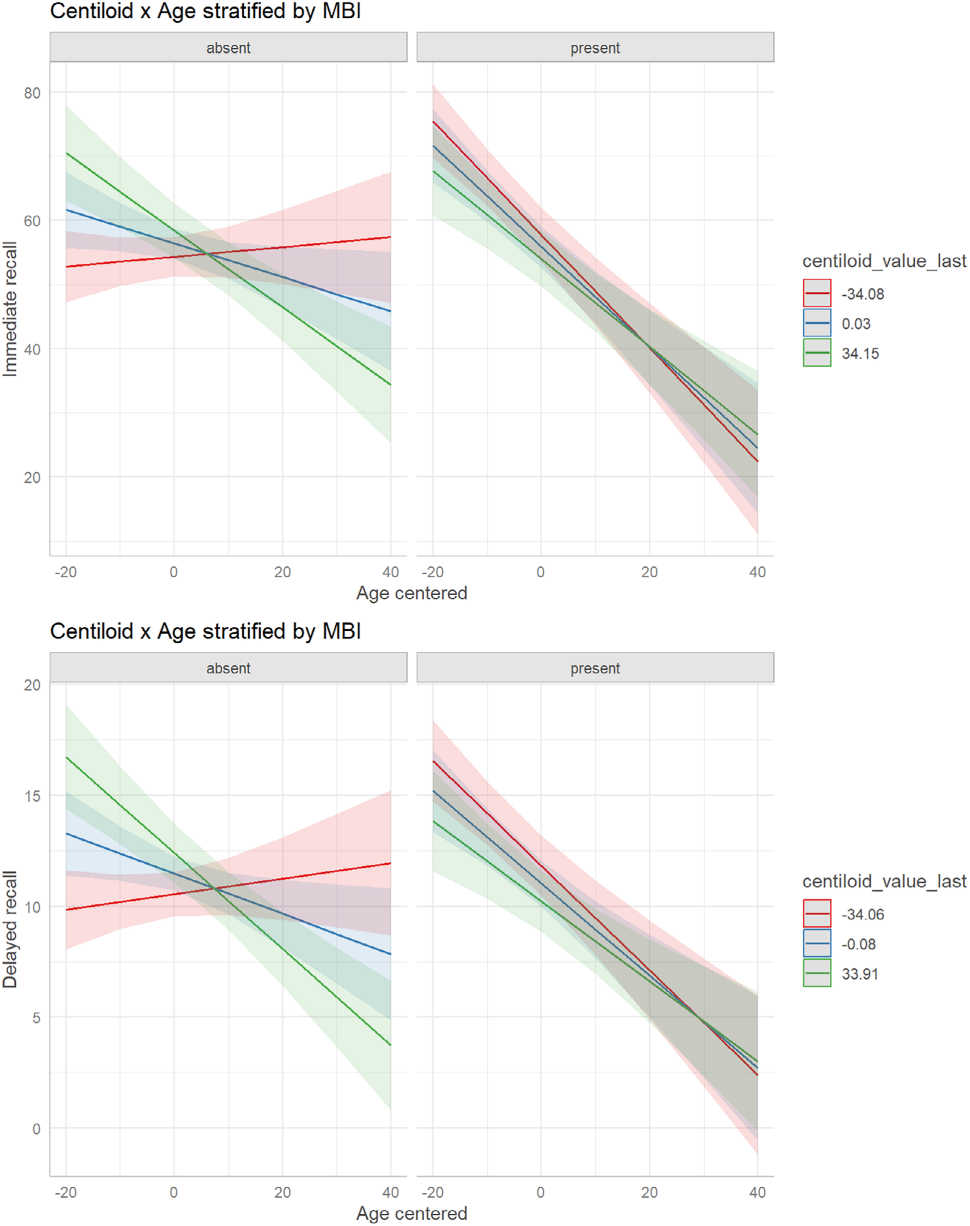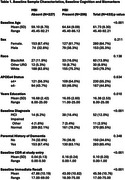# Mild Behavioral Impairment is associated with centiloid concentration and cognitive trajectory

**DOI:** 10.1002/alz70857_104563

**Published:** 2025-12-25

**Authors:** Barbara L. Fischer, Carol A. Van Hulle, Derek L. Norton, Shenikqua Bouges, Gilda E. Ennis, Mary F. Wyman, Diane Carol Gooding, Lauren W. Y. McLester‐Davis, Megan L. Zuelsdorff, Tobey J. Betthauser, Henry Gilreath Stephenson, Sterling C Johnson, Sanjay Asthana, Carey E. Gleason

**Affiliations:** ^1^ Department of Neurology University of Wisconsin‐Madison School of Medicine and Public Health, Madison, WI, USA; ^2^ Wisconsin Alzheimer's Disease Research Center, University of Wisconsin School of Medicine and Public Health, Madison, WI, USA; ^3^ School of Medicine and Public Health, University of Wisconsin‐Madison, Madison, WI, USA; ^4^ Department of Biostatistics and Medical Informatics, University of Wisconsin‐Madison, Madison, WI, USA; ^5^ Madison VA GRECC, William S. Middleton Memorial Hospital, Madison, WI, USA; ^6^ Division of Geriatrics and Gerontology, University of Wisconsin‐Madison, School of Medicine and Public Health, Madison, WI, USA; ^7^ Wisconsin Alzheimer's Institute, University of Wisconsin ‐ Madison School of Medicine and Public Health, Madison, WI, USA; ^8^ Division of Geriatrics and Gerontology, Department of Medicine, University of Wisconsin‐Madison, School of Medicine & Public Health, Madison, WI, USA; ^9^ Department of Psychiatry, University of Wisconsin‐Madison, School of Medicine and Public Health, Madison, WI, USA; ^10^ Department of Psychology, University of Wisconsin‐Madison, College of Letters & Science, Madison, WI, USA; ^11^ Wisconsin Alzheimer's Disease Research Center, MADISON, WI, USA; ^12^ Wisconsin Alzheimer's Disease Research Center, University of Wisconsin‐Madison, SMPH, Madison, WI, USA; ^13^ University of Wisconsin School of Nursing, Madison, WI, USA; ^14^ Wisconsin Alzheimer's Disease Research Center, School of Medicine and Public Health, University of Wisconsin‐Madison, Madison, WI, USA; ^15^ University of Wisconsin‐Madison, Madison, WI, USA; ^16^ Alzheimer's Disease Research Center, University of Wisconsin‐Madison, Madison, WI, USA; ^17^ Department of Medicine, University of Wisconsin‐Madison School of Medicine and Public Health, Madison, WI, USA; ^18^ Geriatric Research, Education and Clinical Center (GRECC), William S. Middleton Memorial Veterans Hospital, Madison, WI, USA; ^19^ Alzheimer's Disease Research Center, University of Wisconsin‐Madison School of Medicine and Public Health, Madison, WI, USA

## Abstract

**Background:**

Support for the association between Mild Behavioral Impairment (MBI) and cognitive decline continues to emerge, but mechanisms remain unclear, particularly in racially diverse cohorts. We investigated relationships between MBI, cognition and six different biomarkers among a richly characterized sample of white and African American (AA) middle aged and older adults. AA participants were enrolled in African Americans Fighting Alzheimer's in Midlife (AA‐FAIM), an ancillary study of the Wisconsin Disease Research Center's (WADRC) Clinical Core and the Wisconsin Registry for Alzheimer's Prevention (WRAP).

**Method:**

Participants enrolled in the WADRC were included in analyses if they were without dementia, had an available Neuropsychiatric Inventory Questionnaire (NPI‐Q) from at least two consecutive visits and one of three biomarker modalities: Hippocampal volume derived from MRI (*n* = 312), centiloid concentration derived from [^11^PiB‐C]‐PET (*n* = 147) or ptau217 pg/mL, Aβ42/40 ratio, glial fibrillary acidic protein pg/mL (GFAP), and Neurofilimant light pg/mL (Nfl) measured in plasma (*n* = 323). We used mixed effects linear models to test if MBI impacts the association between biomarker and cognitive trajectories (i.e. MBI*Biomarker*Time). Cox proportional hazard models were used to assess associations between age to first global CDR >0 occurrence and baseline MBI, most recent biomarker value, and the interaction between the two. Cognitive outcomes included measures of speed, mental flexibility and memory.

**Result:**

There were no significant 3‐way interactions between MBI and HPV, ptau217, Aβ42/40, NfL or GFAP. Individuals with MBI exhibited steeper declines in all cognitive outcomes. Multiple significant 2‐way associations emerged between *p*‐tau217, NfL and GFAP and cognitive decline. After correcting for multiple comparisons, higher centiloid values significantly associated with steeper declines in memory. Cox proportional hazard models yielded no significant interactions between any biomarkers and MBI. Baseline MBI significantly associated with time to CDR>0 in GFAP and centiloid models, although potential overfitting limits interpretability.

**Conclusion:**

Our findings provide preliminary support for the notion that MBI affects the relationship between specific disease biomarkers and cognitive decline, with evidence of this in our centiloid results. Centiloid concentration, a standardized measure of beta amyloid‐PET, may represent a particularly sensitive marker of early, stable brain changes associated with MBI in this racially diverse cohort.